# Screening and Identification of Lassa Virus Entry Inhibitors from an FDA-Approved Drug Library

**DOI:** 10.1128/JVI.00954-18

**Published:** 2018-07-31

**Authors:** Peilin Wang, Yang Liu, Guangshun Zhang, Shaobo Wang, Jiao Guo, Junyuan Cao, Xiaoying Jia, Leike Zhang, Gengfu Xiao, Wei Wang

**Affiliations:** a University of the Chinese Academy of Sciences, Beijing, China; b College of Pharmacy and State Key Laboratory of Medicinal Chemical Biology, Nankai University, Tianjin, China; c State Key Laboratory of Virology, Wuhan Institute of Virology, Chinese Academy of Sciences, Wuhan, China; University of Kentucky College of Medicine

**Keywords:** Lassa virus (LASV), glycoprotein complex (GPC), lacidipine, phenothrin, membrane fusion

## Abstract

Lassa virus (LASV) belongs to the Mammarenavirus genus (family Arenaviridae) and causes severe hemorrhagic fever in humans. At present, there are no Food and Drug Administration (FDA)-approved drugs or vaccines specific for LASV. Here, high-throughput screening of an FDA-approved drug library was performed against LASV entry by using pseudotype virus bearing LASV envelope glycoprotein (GPC). Two hit compounds, lacidipine and phenothrin, were identified as LASV entry inhibitors in the micromolar range. A mechanistic study revealed that both compounds inhibited LASV entry by blocking low-pH-induced membrane fusion. Accordingly, lacidipine showed virucidal effects on the pseudotype virus of LASV. Adaptive mutant analyses demonstrated that replacement of T40, located in the ectodomain of the stable-signal peptide (SSP), with lysine (K) conferred LASV resistance to lacidipine. Furthermore, lacidipine showed antiviral activity against LASV, the closely related Mopeia virus (MOPV), and the New World arenavirus Guanarito virus (GTOV). Drug-resistant variants indicated that V36M in the ectodomain of the SSP mutant and V436A in the transmembrane domain of the GP2 mutant conferred GTOV resistance to lacidipine, suggesting the interface between SSP and GP2 is the target of lacidipine. This study shows that lacidipine is a candidate for LASV therapy, reinforcing the notion that the SSP-GP2 interface provides an entry-targeted platform for arenavirus inhibitor design.

**IMPORTANCE** Currently, there is no approved therapy to treat Lassa fever; therefore, repurposing of approved drugs will accelerate the development of a therapeutic stratagem. In this study, we screened an FDA-approved library of drugs and identified two compounds, lacidipine and phenothrin, which inhibited Lassa virus entry by blocking low-pH-induced membrane fusion. Additionally, both compounds extended their inhibition against the entry of Guanarito virus, and the viral targets were identified as the SSP-GP2 interface.

## Abstract (precis)

Currently, there is no approved therapy to treat Lassa fever; therefore, repurposing of approved drugs will accelerate the development of a therapeutic stratagem. In this study, we screened an FDA-approved library of drugs and identified two compounds, lacidipine and phenothrin, which inhibited Lassa virus entry by blocking low-pH-induced membrane fusion. Additionally, both compounds extended their inhibition against the entry of Guanarito virus, and the viral targets were identified as the SSP-GP2 interface.

## INTRODUCTION

Lassa virus (LASV) is an enveloped, negative-sense, bisegmented RNA virus belonging to the Mammarenavirus genus (family Arenaviridae) (1). Mammarenaviruses consist of 35 unique species currently recognized by the International Committee on Taxonomy of Viruses. The original classification of mammarenaviruses, based mainly on virus genetics, serology, antigenic properties, and geographical relationships, divided them into New World (NW) and Old World (OW) mammarenaviruses (2). The OW LASV and Lujo virus (LUJV), as well as some NW mammarenaviruses, including the Junín virus (JUNV), Machupo virus (MACV), Guanarito virus (GTOV), and Sabiá virus (SABV), are known to cause severe hemorrhagic fever and are listed as biosafety level 4 (BSL-4) agents (3, 4). LASV infections cause about 300,000 cases of Lassa fever per year, and the mortality rate in hospitals is generally 15 to 30% (5). At the beginning of this year, a Lassa fever outbreak was reported in Nigeria. From 1 January to 18 March 2018, 376 confirmed cases and 95 deaths have been reported (6).

The LASV RNA genome encodes the viral polymerase, nucleoprotein, matrix protein (Z), and glycoprotein complex (GPC). GPC is synthesized as a polypeptide precursor that is sequentially cleaved by signal peptidase and the cellular protease subtilisin kexin isozyme-1/site-1 protease to generate the three subunits of the mature complex: the retained stable-signal peptide (SSP), the receptor-binding subunit GP1, and the membrane fusion subunit GP2 (7–10). The highly conserved arenavirus SSPs contain 58 amino acids that span the membrane twice, with 8 amino acids in the ectodomain, playing essential roles in GPC maturation and GPC-mediated membrane fusion (11–17). LASV utilizes α-dystroglycan (α-DG) as a primary receptor, and successful infections require the receptor switch to lysosome-associated membrane protein 1 (18–20).

To date, no vaccines or specific antiviral agents against LASV are available. Therapy strategies are limited to the administration of ribavirin in the early course of the illness (21). To address this issue, we screened an FDA-approved drug library of 1,018 compounds. The approved drugs have been intensively investigated for safety, pharmacokinetics, and targets; therefore, screening approved drugs for repurposing will increase the speed of discovery and development for treatment (22, 23). Drugs targeting viral entry can block replication and spread at an early stage. Since studies of LASV require BSL-4 equipment, we utilized a LASV GPC pseudotype vesicular stomatitis virus (VSV) containing a Renilla luciferase (Rluc) reporter gene for high-throughput screening (HTS) of LASV entry inhibitors, which can be performed in a BSL-2 facility. After three rounds of screening, lacidipine and phenothrin were identified to be highly effective against LASV entry. The hit compounds identified in this study offer potential new therapies to treat arenavirus infections and disease.

(Part of this study was presented in 2017 at the 17th International Congress of Virology, Singapore.)

## RESULTS

### Screening of an FDA-approved drug library for inhibitors of LASV entry.

To perform high-throughput screening under BSL-2 conditions, we generated a pseudotype virus bearing LASV GPC (designated LASVpv) for HTS of entry inhibitors (24). The HTS assay conditions, including the seeding cell density and LASVpv infective dose, were optimized at 1 × 10^4^ cells and 1 × 10^2^ PFU per 96-well plate, respectively. Under the optimized conditions, the signal-to-basal (S/B) ratio, coefficient of variation (CV), and Z′ factor were 41,770, 11.9%, and 0.62, respectively, demonstrating that the assay was promising for large-scale screening of inhibitors.

**FIG 1:**
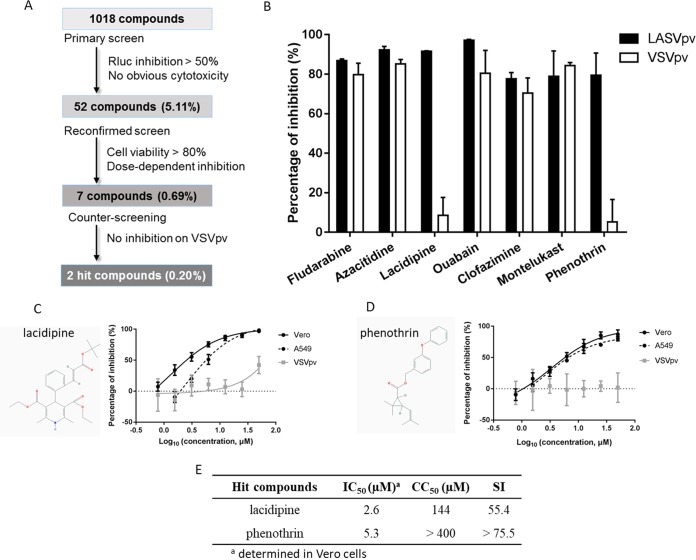
High-throughput screening (HTS) for inhibitors of Lassa virus (LASV) entry from a Food and Drug Administration (FDA)-approved drug library. (A) HTS assay flowchart. (B) Counterscreening of the 7 selected compounds. Vero cells were seeded at a density of 1 × 10^4^ cells per well in 96-well plates. After incubating overnight, cells were treated in duplicate with each compound (25 μM); 1 h later, cells were infected with LASVpv (MOI, 0.01), and the supernatant was removed 1 h postinfection. The infected cells were lysed 23 h later, and the luciferase activities were measured. (C and D) Dose-response curves of lacidipine (C) and phenothrin (D) for inhibiting LASVpv infection. Insets show the structure of each drug. (E) IC_50_, CC_50_, and SI values for lacidipine and phenothrin.

The HTS schematic is depicted in Fig. 1A. Inhibitors were defined as prime candidates, with inhibition of >50% and no apparent cytotoxicity in duplicate wells at a concentration of 10 μM. Of the 1,018 tested compounds, 52 (5.11%) were considered prime candidates. A screening to reconfirm the results was then carried out using these prime candidates over a broader concentration range (3.125 to 50.0 μM). Seven compounds (0.69%) were selected based on their concentration-dependent inhibitory effects and a cell viability of >80%. Subsequently, these 7 compounds were subjected to counterscreening by utilizing VSVpv to rule out inhibitors of VSV genome replication and Rluc. Using these criteria, 2 hits, lacidipine and phenothrin, were selected with specific inhibition against LASV GPC activity, while the other 5 compounds were eliminated (Fig. 1B). Lacidipine is a dihydropyridine voltage-gated Ca^2+^ channel antagonist, while phenothrin is a synthetic pyrethroid used for aerosol insecticides. We evaluated the 50% inhibitory concentration (IC_50_) and 50% cytotoxic concentration (CC_50_) of both hit compounds. Both lacidipine and phenothrin exhibited dose-dependent inhibition of LASVpv infections. The HTS assay was conducted on Vero cells. The inhibitory effects were confirmed by using the A549 human epithelial cell line; epithelial cells are important targets of infection *in vivo*, suggesting these compounds are potentially useful in the treatment of human infections (Fig. 1C and D). The selective index (SI, the ratio of the CC_50_ to the IC_50_) for lacidipine was 55.4, while that for phenothrin was >75.5 (Fig. 1E). The CC_50_ values for the 2 hit compounds were similar to those previously published for diverse cell systems; however, they were determined using different toxicity assays (25). To validate the antiviral effects, lacidipine and phenothrin were purchased from other commercial sources and tested; the cytotoxic and antiviral effects were similar to the results of our primary screening.

### Lacidipine and phenothrin inhibit GPC-mediated membrane fusion.

**FIG 2:**
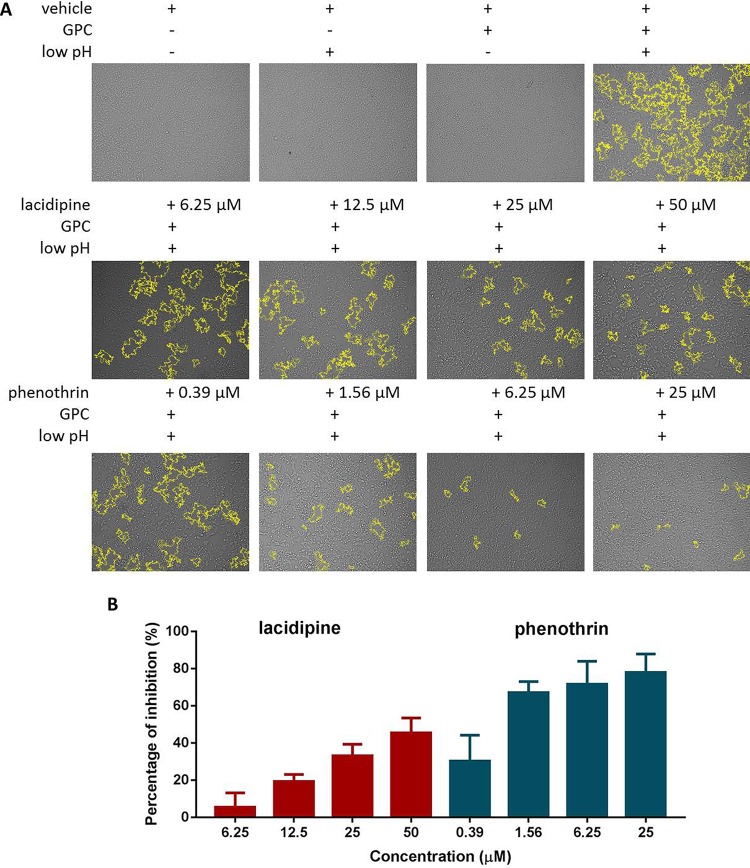
Lacidipine and phenothrin inhibited GPC-mediated membrane fusion. (A) 293T cells were transfected with pCAGGS-LASV GPC or the empty pCAGGS; 24 h later, the compounds or vehicle (DMSO) was added for 1 h followed by treatment with acidified (pH 5.0) DMEM for 15 min. The cells were then placed in neutral pH DMEM. Syncytium formation was visualized after 1 h using light microcopy. Images are representative fields from 4 or 5 independent experiments. Boundaries of syncytia were detected using ImageJ and are outlined in yellow. (B) A dual-luciferase assay was used to quantitatively evaluate the inhibitory activities of lacidipine and phenothrin against membrane fusion. 293T cells transfected with both pCAGGS-LASV GPC and pCAGT7 were cocultured at a ratio of 3:1 with targeted cells transfected with pT7EMCVLuc together with the pRL-CMV control vector. The cell fusion activity was quantitatively determined by measuring firefly luciferase activity and standardized with Rluc activity. Data are presented as means ± standard deviations (SDs) for 4 independent experiments.

Arenavirus GPCs have a unique structure in which the cleaved SSP is retained and noncovalently associates with GP2; many arenavirus entry inhibitors have been shown to bind and stabilize the prefusion forms of GPC to prevent membrane fusion (26–28); therefore, we asked whether these 2 hit compounds act via a similar mechanism. To address this, 293T cells transfected with GPC were incubated with either compound and subjected to a low-pH pulse to promote fusion. As shown in Fig. 2A, a low pH in the GPC-transfected cells induced obvious membrane fusion, whereas a neutral pH had no effect. Phenothrin significantly inhibited syncytium formation at all tested concentrations, while minor inhibition was observed in the lacidipine-treated group.

To further quantitatively evaluate the inhibitory activities, fusion efficacy was determined using a dual-luciferase assay (27, 29, 30). As shown in Fig. 2B, both compounds exhibited a dose-dependent inhibition of GPC-mediated membrane fusion, suggesting that both compounds inhibit GPC conformational changes induced by an acidic environment. Notably, the inhibition caused by phenothrin could reach ∼80% at 25 μM, while the inhibition by lacidipine was ∼50% even at 50 μM, which corresponds to nearly 20 times the IC_50_. It might be due to the differences inherent in the two assays (26, 31–33). No higher concentration of lacidipine was utilized because of its cytotoxicity and insolubility. Together, these results show that both compounds prominently inhibit GPC-mediated membrane fusion.

### Lacidipine exerts a virucidal effect on LASVpv.

**FIG 3:**
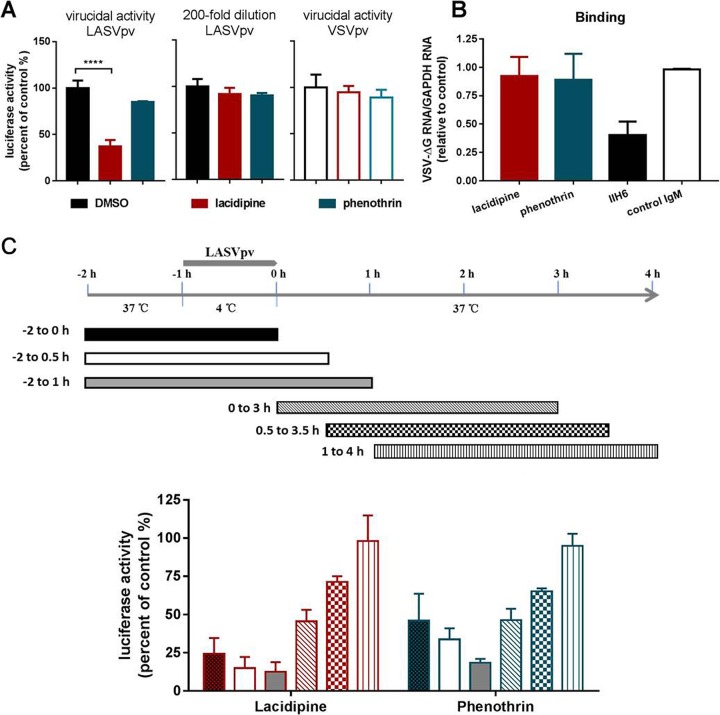
Effects of lacidipine and phenothrin on different stages of Lassa virus (LASV) entry. (A) Results of the virucidal assay. (Left) LASVpv at an MOI of 2 were incubated with DMSO or compounds (25 μM for 1 h) and then diluted 200-fold and added to cells. ****, *P* < 0.0001. (Middle) LASVpv at an MOI of 0.01 were incubated with DMSO or compounds (0.125 μM for 1 h) and then added to cells. (Right) VSVpv at an MOI of 2 was incubated with DMSO or compound (25 μM for 1 h) and then diluted 200-fold and added to cells. Luciferase activities were determined 24 h later. (B) Effects of compounds on LASVpv binding. Vero cells were preincubated with compounds (50 μM) or vehicle at 37°C for 1 h, followed by incubation with LASVpv (MOI, 10) in the presence or absence of compounds at 4°C for an additional 1 h. After extensively washing with cold PBS, the bound virus was quantified via RT-qPCR. The preincubation treatments with MAb IIH6 (200 μg/ml) and an unrelated mouse IgM were conducted at 4°C for 2 h. (C) Time-of-addition assay. The experimental timeline is shown in the upper panel. Vero cells were infected with LASVpv (MOI, 0.01) at 4°C for 1 h and then washed with PBS; the temperature was then increased to 37°C in the presence of lacidipine (10 μM) or phenothrin (25 μM) for the indicated times. Data are presented as the means ± SDs for 3 independent experiments.

Lacidipine and phenothrin inhibited GPC-mediated membrane fusion; therefore, we asked whether the compounds could bind to GPC and exert a virucidal effect. To test this, LASVpv was mixed with each compound for 1 h; the mixture was then diluted 200-fold to the noninhibitory concentration of 0.125 μM, about 1/20 of the IC_50_, and added to the cells for 1 h (34). As shown in Fig. 3A, luciferase activity was not suppressed in the phenothrin group, while a reduction of >60% was observed in the lacidipine group, indicating that lacidipine exhibited a virucidal effect on LASVpv. Considering that lacidipine is a highly lipophilic molecule with a high membrane partition coefficient, the accumulation of the compound in viral membrane might facilitate the binding to GPC and result in a low off rate (27, 34–36).

We next investigated the inhibitory effects of the compounds on binding, which was initialized when GP1, the receptor binding subunit, recognized the primary receptor α-DG (18). Binding efficacy was evaluated in the absence and presence of the compounds, and no significant decrease in the number of LASVpv particles bound to the cell surface at 4°C was observed in either compound-treated group (Fig. 3B), suggesting that neither compound interferes with the receptor binding subunit GP1 (37, 38). The monoclonal antibody (MAb) IIH6, recognizing a functional glycan epitope on α-DG and competing with LASV binding (37, 39), was used as a positive control, which blocked LASVpv infection at a concentration of 200 μg/ml.

To further confirm compound mechanisms, we studied the inhibition kinetics of lacidipine and phenothrin. The experimental timeline is depicted in Fig. 3C. Pretreatment of cells with lacidipine or phenothrin sharply decreased LASVpv infection even when removed at 30 min postinfection. The addition of compounds 30 min postinfection resulted in a mild inhibitory effect, while the addition of compounds 1 h postinfection had no effect, which was in line with the previous reports that preexposure with the low-off-rate inhibitors was sufficient for inhibition (27, 32). Taken together, we concluded that the early stage of LASVpv infection, especially the membrane fusion, was the sensitive phase of lacidipine.

### The adaptive mutant results in the SSP T40K.

**FIG 4:**
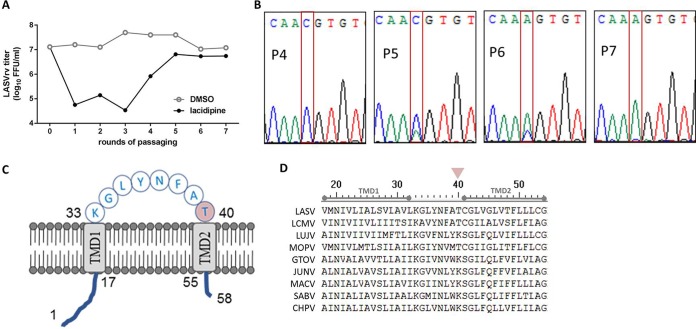
Selection of lacidipine-resistant LASVrv. (A) The adaptive mutant virus was selected by serially passaging LASVrv in the presence of 10 μM lacidipine. In a parallel experiment, LASVrv passaging in vehicle served as a control. After 7 rounds of passaging, no further improvement in resistance was detected, and the selection was terminated. (B) Sequencing chromatograms of P4 to P7 viruses. The red inbox highlights the emergence of the adaptive mutant. (C) Membrane topology of LASV SSP with the T40 location highlighted. (D) Amino acid sequence alignment of the mammarenavirus SSP. The GenBank accession numbers are listed in Materials and Methods.

To identify the viral target of the compounds, we selected an adaptive mutant virus by serially passaging the replication-competed LASVrv in the presence of 10 μM lacidipine and 25 μM phenothrin, corresponding to about IC_85_ values of both compounds. Parallel passaging of LASVrv in dimethyl sulfoxide (DMSO) was used as a control. In the lacidipine-treated group, robust resistance was detected after 7 rounds of passaging (Fig. 4A). We sequenced the lacidipine-resistant LASVrv from passage 3 (P3) to P7 without plaque purification. As shown in Fig. 4B, up to P4, no mutation occurred. Notably, the adaptive mutant emerged from P5 since there were overlapped peaks at the corresponding amino acid position 40 of SSP, in which the higher peak read as “C” resulted in threonine (ACG) and the lower peak read as “A” resulted in lysine (AAG). In P6, the “A” peak grew higher than the “C” peak, indicating that the drug-resistant viruses gradually increased under the pressure of lacidipine. In P7, the “C” peak became lower and was similar to the background noise. The threonine (T)-to-lysine (K) switch was located at the last position of the SSP ectodomain (Fig. 4C and D), which was absent in the DMSO-treated virus. Notably, we have tried to induce phenothrin-resistant LASVrv by gradually increasing the concentration from 25 to 100 μM, i.e., 25 μM for the first 10 passages, 50 μM for the next 7 passages, and 100 μM for the last 3 passages. However, no adaptive mutant was detected, suggesting that phenothrin is less prone to inducing adaptive mutations in the glycoprotein.

### Position 40 in SSP is tolerable for K.

The arenavirus SSP, unlike in other enveloped viruses, is unusually long and retains the GPC as a vital subunit, playing an essential role in glycoprotein maturation and GPC-mediated membrane fusion (12, 16, 38). The 58-amino-acid SSP contains two hydrophobic domains linked by an 8-amino-acid ectodomain loop that interacts with the proximal and transmembrane region of GP2 to confer sensitivity of fusion inhibitors (26, 28, 33).

**FIG 5:**
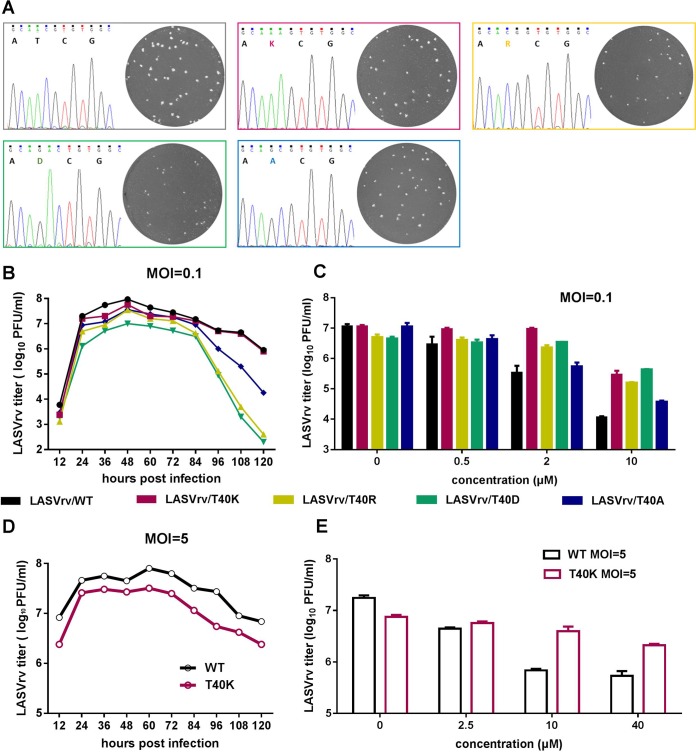
Characterization of lacidipine-resistant LASVrv. (A) Sequencing chromatograms of the WT and recombinant viruses with plaque morphology of each virus shown in insets. (B) Growth kinetics of the recombinant viruses with different T40 mutations. Vero cells were infected with recombinant viruses (MOI = 0.1) for 1 h. The supernatants were collected at indicated time points postinfection and assayed for the viral titer. (C) Resistant activities of the recombinant viruses (MOI = 0.1) to lacidipine. (D) Growth kinetics of WT and T40K LASVrv with an MOI of 5. (E) Resistant activities of the WT and T40K LASVrv (MOI = 5) to lacidipine. Data are presented as means ± SD from 2 independent experiments.

To confirm that the T40K mutation conferred lacidipine resistance and to investigate the role of T40 in SSP function and lacidipine inhibition of LASV entry, we produced recombinant viruses with T40K, T40R, T40D, or T40A mutations by introducing the desired mutations into the GPC gene and generating mutant viruses (Fig. 5A). To investigate the biological properties of the mutant viruses, we first examined the growth kinetics of the rescued viruses. As shown in Fig. 5B, all the viruses with a multiplicity of infection (MOI) of 0.1 caused an accumulation of infectious virions that reached the highest titer at 48 h postinfection (p.i.). When infected with a high MOI (of 5), the titers of both wild-type (WT) and T40K viruses rose to 10^6^ to 10^7^ PFU/ml in 12 h and these levels lasted for at least 120 h (Fig. 5D). Infection of T40K mutant viruses resulted in growth curves similar to those of the WT virus with either low or high MOI, while T40R and T40D mutants produced less virus after 18 to 36 h. Plaque morphology analyses revealed that the T40K plaques were similar to the WT plaques, whereas T40R and T40D plaques were smaller and T40A plaques were midrange. These results suggested that T40R and T40D mutant viruses are attenuated and position 40 in SSP is tolerable for K.

### T40K mutation conferring resistance to lacidipine.

We next investigated the sensitivity of the four mutant viruses to lacidipine. As shown in Fig. 5C, T40K, T40R, and T40D mutant viruses conferred resistance to lacidipine. Lacidipine at 10 μM efficiently inhibited LASVrv WT infection by reducing the viral yields by 3 log units. In contrast, T40K, T40R, and T40D mutant viruses were insensitive to lacidipine, with the viral titer decreasing slightly less than 1 log unit, while the T40A mutant virus showed no resistance. Similar results were obtained with infections at a high MOI (Fig. 5E).

Taken together, these results indicated that the T40 mutant was not only critical in conferring lacidipine sensitivity but also important for LASV infectivity. Replacement of T with K conferred resistance to lacidipine without apparent loss of growth, while substitution of a small nonpolar amino acid (A) did not affect lacidipine sensitivity. Other positively charged amino acids (R) or negatively charged amino acids (D) resulted in the mutant viruses propagating more slowly than the WT virus.

### Lacidipine affects entry of other arenaviruses.

**FIG 6:**
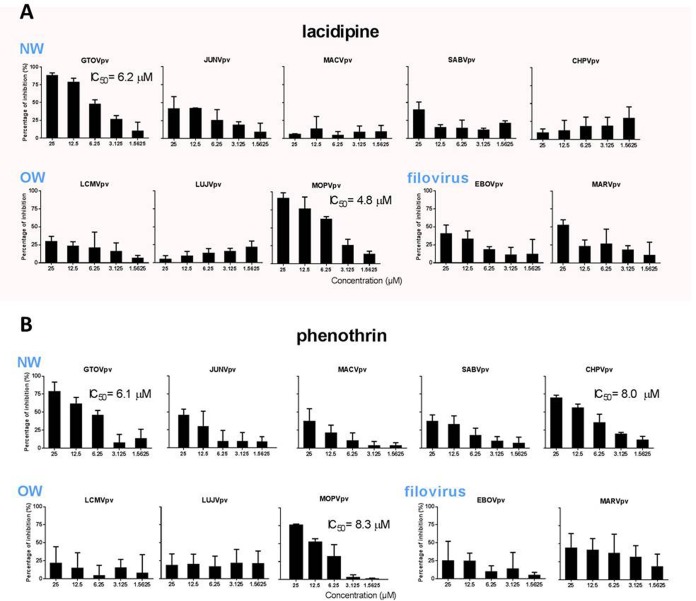
Broad-spectrum antiviral activity of the hit compounds against different mammarenaviruses and filoviruses. Vero cells were incubated in the absence and presence of lacidipine (A) or phenothrin (B). After 1 h, the pseudotypes of GTOV, JUNV, MACV, SABV, CHPV, LCMV, LUJV, MOPV, EBOV, and MARV were added at an MOI of 0.01. The supernatant was removed 1 h later, and the cell lysates were assessed for luciferase activity after 23 h. Data are presented as means ± SD from 3 independent experiments.

T40 is conserved in OW viruses, except for LUJV, whereas K40 is similarly conserved (K or R) in NW viruses; therefore, we investigated the effects of lacidipine on the entry of other pathogenic pseudotype arenaviruses, such as OW viruses (including lymphocytic choriomeningitis virus [LCMV], LUJV, and the closely related Mopeia virus [MOPV]) and NW viruses (including JUNV, MACV, GTOV, SABV, and Chapare virus [CHPV]) using the pseudotype viruses. As shown in Fig. 6A, all viruses mentioned above remained unaffected, except for GTOVpv and MOPVpv, which exhibited dose-dependent inhibition with IC_50_s of 6.2 and 4.8 μM, respectively. We also investigated the broad-spectrum antiviral activity of phenothrin. It was shown that phenothrin dose dependently inhibited the entry of GTOVpv, MOPVpv, and CHPVpv, with IC_50_s of 6.1, 8.3, and 8.0 μM, respectively. Phenothrin had a less powerful effect on the entry of JUNV, MACV, and SABV, since the percentage of inhibition was less than 50% at the highest tested concentration (Fig. 6B).

To further assess the role of lacidipine and phenothrin on other class I fusion proteins, we utilized pseudotype Ebola virus (EBOV) and Marburg virus (MARV). It is important to note that neither lacidipine nor phenothrin treatment robustly inhibited the entry of EBOVpv and MARVpv (Fig. 6), indicating a lacidipine- or phenothrin-related interaction with GPC of arenaviruses.

### Selection of lacidipine-resistant GTOVrv.

**FIG 7:**
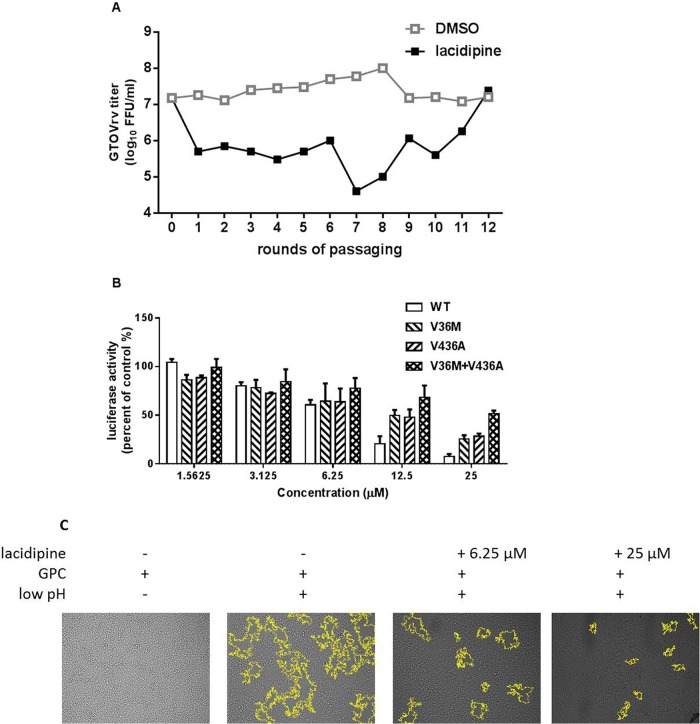
Selection of lacidipine-resistant GTOVrv. (A) The adaptive mutant virus was selected by serially passaging GTOVrv in the presence of 10 μM lacidipine. GTOVrv passaging in vehicle served as a control in parallel. After 12 rounds of passaging, no further improvement in resistance was detected and the selection was terminated. (B) Resistant activity of the GTOVpv with the adaptive mutants to lacidipine. Data are presented as means ± SD from 2 independent experiments. (C) Lacidipine inhibited GTOV GPC-mediated membrane fusion. 293T cells were transfected with pCAGGS-GTOV GPC; 24 h later, lacidipine was added for 1 h, followed by treatment with acidified (pH 5.0) DMEM for 15 min. Syncytium formation was visualized after 1 h by using light microcopy. Images are representative fields from 3 independent experiments. Boundaries of syncytia were detected using ImageJ and are outlined in yellow.

GTOV possesses a K at position 40 of SSP; therefore, we assumed that an amino acid other than K40 contributed to the sensitivity of GTOV to lacidipine. We further determined the viral target by selecting the resistant GTOVrv in the presence of 10 μM lacidipine. After 12 rounds, 2 amino acid substitutions, V36M in the ectodomain of SSP and V436A in the transmembrane domain of GP2, were observed in the resistant virus (Fig. 7A). To investigate the functional significance of these residues, pseudotype GTOV containing the mutants were used to evaluate lacidipine sensitivity. GTOVpv was much less sensitive when either the V36M or V436A mutant was generated, and lacidipine sensitivity was further reduced when both sites were changed (Fig. 7B). Moreover, it was determined that lacidipine-mediated inhibition of GTOVpv was also associated with inhibition of the GPC-mediated membrane fusion (Fig. 7C). Together, these results indicated that lacidipine inhibited LASV and GTOV entry by targeting the SSP-GP2 interface, stabilizing the prefusion structure of GPC, and thus preventing membrane fusion.

## DISCUSSION

In this study, we screened an FDA-approved drug library and identified 2 hit compounds, lacidipine and phenothrin, which prohibited the entry step of pseudotype and recombinant LASV infection. Lacidipine is a lipophilic dihydropyridine calcium antagonist. Since calcium channels proved to be a therapeutic target for other enveloped viruses and calcium inhibitors showed promising effects on the entry of the closely related JUNV and EBOV (22, 40–42), we investigated whether lacidipine inhibited LASV entry by acting as a calcium inhibitor. To address this, we first reviewed all 22 calcium inhibitors included in the current FDA drug library. It was shown that in addition to lacidipine, only two calcium inhibitors, diltiazem and cilnidipine, moderately inhibited LASVpv entry to <70% inhibition at the highest tested concentration (20 μM). Results were consistent with those previously published for other calcium inhibitors, such as verapamil, nifedipine, and tetrandrine, exhibiting weak or no inhibition on recombinant and pseudotype LASV infection (34, 43). Although in another paper it was reported that verapamil and nifedipine could inhibit pseudotype LASV entry, the suppression level in LASV GPC was much lower than in JUNV GPC (40). Together, these results suggested that calcium inhibitors do not effectively block LASV entry as observed for other enveloped viruses. Although it was not necessary to exclude the calcium inhibitor effect possessed by lacidipine, it acted mainly as a fusion inhibitor by interacting with the LASV GPC.

Lacidipine is a highly lipophilic compound and is proved to be deeply embedded in the membrane's hydrocarbon core (36), which might explain the long clinical half-life (13 to 19 h) of lacidipine and also might account for our results showing that lacidipine has a virucidal effect while phenothrin does not. The accumulation of lacidipine in viral membrane might contribute to the binding of the compound with LASV GPC.

To further elucidate the underlying mechanisms associated with prevention of LASV entry via lacidipine, we characterized the viral target of lacidipine by serially passaging LASVrv in the presence of lacidipine. The adaptive mutant was found to be located in the interface of SSP and GP2, which was proven to be the target for a range of fusion inhibitors identified in previous drug screens (8, 12, 16, 28, 32, 33, 38, 41, 42, 44–46). In particular, we recovered an interesting resistance mutation, T40K, in the ectodomain of SSP. Among the 8 amino acid residues in the ectodomain of SSP, the absolutely conserved K33 and N37 are well studied and have proven to be essential in GPC maturation, fusion, and the sensitivity to other fusion inhibitors (12, 26, 47). T40 and K40 are relatively conserved in OW and NW viruses, respectively. However, in the broad-spectrum antiviral investigation, only MOPV and GTOV were sensitive to lacidipine, suggesting that although the SSP-GP2 interfaces of arenavirus GPC are similar in structure and function, the interplay between SSP and GP2 might involve a multiple sequential correlation and is not limited at the point-to-point subinterface. Substitutions of some residues, such as adaptive mutants confirmed in the current work, had little effect on the structure and function of GPC, or the effect might be compensated by the neighboring interaction. However, these resistant mutants might play essential roles in the interaction, directly or indirectly, with a distinct fusion inhibitor, and this interaction was exclusive and could hardly be compensated or replaced by neighboring residues. Moreover, the inhibitor sensitivity depends on accessibility of the interface to the inhibitor as well as the stability of the inhibitor-GPC complex. As mentioned above, the high lipotropy of lacidipine might contribute to the stability of the complex.

We also identified phenothrin as a hit compound via HTS; phenothrin is a pyrethroid usually used in pesticide products and is effective in inhibiting LASVpv entry. We demonstrated that phenothrin has activity against MOPVpv, GTOVpv, and CHPVpv, with IC_50_s lower than 10 μM. In our study, the phenothrin-resistant viruses failed to generate even under a high selection pressure of up to 100 μM, suggesting this compound might interact with glycoprotein with a relatively lower affinity or a faster off-rate than lacidipine. In line with this, phenothrin exhibited little effect in the virucidal assay.

Until now, the usage of phenothrin was limited to insecticide products. The low toxicity of phenothrin is due to its limited absorption and rapid biodegradation (http://www.who.int/iris/handle/10665/41127). These characteristics of phenothrin might restrict its development for the treatment of infectious diseases for humans. For lacidipine, the low toxicity (the 50% oral lethal dose in rabbit being 3,200 mg/kg of body weight) and the long clinical half-life, together with the high membrane partition coefficient (48, 49), are great benefits indicating that it can be regarded as a viable lead compound for further development as antiviral drug. Although the IC_50_ reported here is higher than the peak concentrations in plasma (3.5 to 12.5 nM) following a single-dose oral administration of 4 mg in treatment of hypertension (48), the method and dosage of administration could be improved to meet the effective level for antiviral treatment. Moreover, use of a combination of lacidipine with replication inhibitors might improve its therapeutic efficacy, reduce the toxicity, and reduce the risk of resistance development. Lacidipine, phenothrin, and the reported LASV-specific entry inhibitor ST-161 are structurally distinct compounds with no obvious common characteristic (26, 31). Understanding the structure-function relationship of these compounds might provide a novel stratagem in the drug design for arenavirus treatment.

## MATERIALS AND METHODS

### Cells and viruses.

BHK-21, HEK 293T, Vero, HeLa, and A549 cells were cultured in Dulbecco's modified Eagle's medium (DMEM; HyClone, Logan, UT, USA) supplemented with 10% fetal bovine serum (Gibco, Grand Island, NY, USA). The pseudotype VSV bearing the GPC of LASV (strain Josiah, GenBank accession number HQ688673.1), LCMV (strain Armstrong, AY847350.1), LUJV (NC_012776.1), MOPV (AY772170.1), GTOV (NC_005077.1), JUNV (strain XJ13, NC_005081.1), MACV (strain Carvallo, NC_005078.1), SABV (U41071.1), CHPV (NC_010562.1), EBOV (strain Mayinga, EU224440.2), and MARV (YP_001531156.1) were generated as previously reported (50–52). 293T cells transfected with pCAGGS-GPC were infected with pseudotype VSV (described below) in which the G gene was replaced with the luciferase gene at an MOI of 0.1 for 2 h. The culture supernatants were harvested 24 h later, centrifuged to remove cell debris, and stored at −80°C. The recombinant VSV expressing the GPC of LASV and GTOV were generated as described previously (53, 54). The plasmid used for construction recombinant virus was pVSVΔG-eGFP (where eGFP is enhanced green fluorescent protein; plasmid 31842; Addgene). GPC was cloned into the ΔG site, and the construct was designated pVSVΔG-eGFP-GPC. BHK-21 cells in 6-well plates were infected with a recombinant vaccinia virus (vTF7-3) encoding T7 RNA polymerase at an MOI of 5. Forty-five minutes later, cells were transfected with 11 μg of mixed plasmids with a 5:3:5:8:1 ratio of pVSVΔG-eGFP-GPC (pVSVΔG-Rluc for generating pseudotype VSV), pBS-N, pBS-P, pBS-G, and pBS-L. After 48 h, the supernatants were filtered to remove the vaccinia virus and inoculated into BHK-21 cells that had been transfected with pCAGGS-VSV G 24 h previously. The pseudotype and recombinant viruses bearing LASV GPC are designated LASVpv and LASVrv, respectively.

The titer of pseudotype virus was measured by infecting BHK-21 cells previously transfected with pCAGGS-VSV G and determined by plaque assay 24 h p.i. The titer of recombinant virus was determined by plaque assay. The titers of LASVpv and LASVrv were 3 × 10^7^/ml and 1.6 × 10^7^/ml, respectively.

### Optimization of HTS assay conditions.

Cell density and MOI were optimized for the HTS assay. Vero cells at different densities (2,500 to 12,500 cells per 96-well plate) were infected at MOIs from 0.001 to 1. The appropriate cell density and the dose for LASVpv were selected by comparing the signal-to-basal ratio, the coefficients of variation, and Z′ values under different conditions as previously described (34, 55). Methyl-beta-cyclodextrin (MβCD; 2 mM) and 0.5% DMSO were used as the positive and negative controls, respectively, for the determination of Z′.

### HTS assay of an FDA-approved compound library.

A library of 1,018 FDA-approved drugs was purchased from Selleck Chemicals (Houston, TX, USA). Compounds were stored as 10 mM stock solutions in DMSO at −80°C until use. The first-round HTS was carried out as shown in Fig. 1A. Briefly, Vero cells were seeded at a density of 1 × 10^4^ cells per well in 96-well plates. After incubating overnight, cells were treated in duplicate with the compounds (10 μM); 1 h later, cells were infected with LASVpv (MOI, 0.01), and the supernatant was removed 1 h postinfection. The infected cells were lysed 23 h later, and luciferase activity was measured using the Rluc assay system (Promega, Madison, WI). Primary candidates were identified using criteria of no apparent cytotoxicity and an average >50% inhibition in duplicate wells and then subsequently rescreened via serial dilution in triplicate plates to evaluate the IC_50_ (GraphPad Prism 6). Dose-dependent inhibition and cell viability of >80%, determined by 3-(4,5-dimethyl-2-thiazolyl)-2,5-diphenyl-2H-tetrazolium bromide (MTT) assay, were the criteria used to select 7 compounds. The 7 compounds were then counterscreened using VSVpv (MOI, 0.01) to rule out that inhibitors acted on VSV genome replication or Rluc activity. Compounds specifically blocking LASV entry were considered hit compounds and were evaluated for the CC_50_ and SI.

### Membrane fusion assay.

293T cells transfected with pCAGGS-LASV GPC or the empty pCAGGS were treated with compounds or vehicle (DMSO) for 1 h, followed by incubation for 15 min with acidified (pH 5.0) medium. The cells were then placed in neutral medium, and syncytium formation was visualized 1 h later via light microscopy. Boundaries of syncytia were automatically traced by using the “versatile wand” tool of ImageJ (56, 57).

For quantification of the luciferase-based fusion assay, 293T cells in 24-well plates transfected with both pCAGGS-LASV GPC (0.25 μg) and plasmids expressing T7 RNA polymerase (pCAGT7, 0.25 μg) were cocultured at a ratio of 3:1 with targeted cells transfected with pT7EMCVLuc (2 μg per well for 6-wells plate) and 0.1 μg pRL-CMV (plasmids used in this assay were kindly provided by Yoshiharu Matsuura, Osaka University, Osaka, Japan). After 12 h of incubation, the compound treatment and pH induction were conducted as described above. Cell fusion activity was quantitatively determined after 24 h by measuring firefly luciferase activity expressed by pT7EMCVLuc and was standardized with Rluc activity expressed by pRL-CMV by using the Dual-Glo luciferase assay (Promega) (27, 29, 30).

### Virucidal assay.

To study the virucidal effects of the compounds, approximately 5 × 10^5^ PFU of LASVpv or VSVpv was incubated with compounds (25 μM) or vehicle at 37°C for 1 h; the mixture was diluted 200-fold to a noninhibitory concentration (MOI, 0.01) to infect Vero cells in a 48-cell plate. The luciferase activity was determined 24 h later as described above.

### Binding assay.

Vero cells were pretreated with 50 μM lacidipine or phenothrin at 37°C for 1 h. As a control, Vero cells were pretreated with 200 μg/ml IIH6 (sc-53987; Santa Cruz) or a control IgM at 4°C for 2 h in parallel (37, 39). Then, the cells were transferred onto ice, and LASVpv (MOI, 10) was added for 1 h. After being washed with cold phosphate-buffered saline (PBS) 3 times, the bound viral particles were quantified via reverse transcriptase quantitative PCR (RT-qPCR) using a specific primer pair to detect the VSVΔG-Rluc (primers 5′-GTAACGGACGAATGTCTCATAA-3′ and 5′-TTTGACTCTCGCCTGATTGTAC-3′). All RNA amplifications were normalized to glyceraldehyde 3-phosphate dehydrogenase (GAPDH) RNA (obtained via PCR with the following primers: 5′-TCCTTGGAGGCCATGTGGGCCAT-3′ and 5′-TGATGACATCAAGAAGGTGGTGAAG-3′).

### Time-of-addition assay.

The experimental timeline is depicted in Fig. 3C. At time −1, Vero cells were infected with LASVpv (MOI, 0.01) at 4°C for 1 h and washed with PBS; the temperature was then increased to 37°C to synchronize the infections. Lacidipine at 10 μM and phenothrin at 25 μM were incubated with the cells for different time courses as shown in Fig. 3C.

### Selection of adaptive mutants.

Drug-resistant viruses were generated by passaging LASVrv on Vero cells in the presence of 10 μM lacidipine or 25 to 100 μM phenothrin. LASVrv was passaged in the presence of 0.5% DMSO in parallel as a control. Passaging in the presence of lacidipine was terminated when no further improvement in resistance was detected. RNA from the resistant viruses was extracted by TRIzol (TaKaRa) and reverse transcribed by using the PrimeScript RT reagent kit (TaKaRa). The GPC segment was amplified and sequenced by using the primers located at ∼100 bp upstream and downstream of GPC: (5′-CCAGCTTCTGAACAATCCCCG-3′ and 5′-GTGGACTTCCATGATTGCTG-3′). Mutant sites were introduced to recover LASVrv as previously described (58). Virus titers and lacidipine sensitivities were determined by means of a plaque assay in Vero cells.
